# In Vitro Assessment of Total Phenolic and Flavonoid Contents, Antioxidant and Photoprotective Activities of Crude Methanolic Extract of Aerial Parts of *Capnophyllum peregrinum* (L.) Lange (Apiaceae) Growing in Algeria

**DOI:** 10.3390/medicines5020026

**Published:** 2018-03-22

**Authors:** Mostefa Lefahal, Nabila Zaabat, Radia Ayad, El hani Makhloufi, Lakhdar Djarri, Merzoug Benahmed, Hocine Laouer, Gema Nieto, Salah Akkal

**Affiliations:** 1 Valorization of Natural Resources, Bioactive Molecules and Biological Analysis Unit, Department of Chemistry, University of Mentouri Constantine, Constantine 25000, Algeria; mlefahal@gmail.com (M.L.); z_nabila2002@yahoo.fr (N.Z.); radia.ayad@yahoo.fr (R.A.); makhloufi_el_hani@yahoo.fr (E.M.); djarri62@gmail.com (L.D.); 2 Department of Chemistry, Faculty of Science, University of Tebessa, Tebessa 12000, Algeria; Riad43200@yahoo.fr; 3 Laboratory of Natural Biological Resources Valorization, Faculty of Sciences, University of Setif, Setif 19000, Algeria; hocine_laouer@yahoo.fr; 4 Department of Food Technology, Nutrition and Food Science, Faculty of veterinary Sciences, University of Murcia, Campus de Espinardo, Espinardo, 30100 Murcia, Spain

**Keywords:** *Capnophyllum peregrinum*, antioxidant activity, photoprotective activity

## Abstract

**Background:** *Capnophyllum peregrinum* (L.) Lange (Apiaceae) is the unique taxon of *capnophyllum* genus in Algerian flora. It has never been investigated in regards to its total phenolic and flavonoid contents and antioxidant and photoprotective activities. **Methods:** *C. peregrinum* aerial parts extracted with absolute methanol. The total flavonoid and phenolic contents of the extract were evaluated to determine their correlation with the antioxidant and photoprotective activities of the extract. **Results:** The methanolic extract demonstrated a significant amount of phenolics and flavonoids (74.06 ± 1.23 mg GAE/g, 44.09 ± 2.13 mg QE/g, respectively) and exhibited good antioxidant activity in different systems, especially in 1,1-Diphenyl-2-picrylhydrazyl (DPPH), reducing power and total antioxidant capacity assays. Furthermore the extract showed high photoprotective activity with the sun protection factor (SPF) value = 35.21 ± 0.18. **Conclusions:** The results of the present study show, that the methanolic extract could be used as a natural sunscreen in pharmaceutics or cosmetic formulations and as a valuable source of natural antioxidants.

## 1. Introduction

Since ancient times, medicinal plants have been used for their medicinal virtues and nutriment values. Presently, the crude extracts from both medicinal and aromatic plants have attracted keen interest in the development and preparation of alternative traditional medicine and food additives [1].

Apiaceae is one of the best-known families of flowering plants, which comprises of about 300–455 genera and 3000–3750 species worldwide [2]. In Algerian flora this family is represented by 55 genera and 130 species [3]. Several Apiaceae members are aromatic plants that are popularly used in medicine and in cooking, such as *Anethum graveolens*, *Angelica archangelica*, *Apium graveolens*, *Carum carvi*, *Coriandrum sativum*, and *Foeniculum vulgare* [4]. *Capnophyllum* Gaertner belongs to the Apiaceae family, which is a genus of small annual herbs, and its name is derived from the Greek (Capnos = smoke, phyllon = leaf) [5]. This genus is represented in the Algerian flora by unique taxon known as *Capnophyllum peregrinum* (L.) Lange [3]. Concerning the uses of this species, it has been mentioned that its roots are used as vegetables [6]. Regarding the phytochemical investigations on this taxon, only one data item has been published so far, indicating the presence of phtalides, such as Z-ligustilide, in its roots [6].

Natural products from plant sources have the ability to reduce oxidative stress by acting as antioxidants [7]. Hence, plants still present a large source of natural antioxidants that could be taken into account for searching and developing the novel drugs. In fact, phenolic compounds from natural sources are well known as radical scavengers, metal chelators, reducing agents, and hydrogen donors. Thus, plants with high levels of polyphenols have attracted significant importance to be used as natural antioxidants [8]. 

The ultraviolet radiation is divided into three categories: UV-A (320–400 nm), UV-B (290–320 nm), and UV-C (200–290 nm). The prolonged exposure to these radiations can generate harmful effects which can cause several skin ailments (in particular, UV-B radiation (290–320 nm)) that can cause erythema, sunburns, DNA damage, and premature aging of skin [9]. They also led to the generation of reactive oxygen species (ROS) [10]. Thus, protecting human skin against UV radiation is evident and the effective protection results in the use of formulations containing solar filters (chemicals that absorb UV radiations) known as sunscreens [11]. Recently, plant extracts with antioxidant properties have been used in phytocosmetic field to be used as sunscreens. In fact, that they comprise ingredients that could inactivate ROS and prevent erythema and premature aging of the skin [12].

To find new natural sources which could be used as natural antioxidants and as sunscreens in pharmaceutical or cosmetic preparations, the main aim of this study was to evaluate the in vitro total phenolic and flavonoid contents, antioxidant, and photoprotective activities of crude methanolic extract of aerial parts of *C. peregrinum* growing in Algeria.

## 2. Materials and Methods 

### 2.1. Plant Materials

*C. peregrinum* aerial parts were collected in May 2015 from Elkala (eastern Algerian). The plant was identified by Prof. Dr. H. Laouer from the Department of biology and plant ecology, Ferhat Abbas University (Setif, Algeria) and a voucher specimen were deposited in the Herbarium of our laboratory. 

#### Preparation of Extracts

*C. peregrinum* aerial parts were dried in a shade at room temperature. A total of 20 g of dried aerial parts were soaked in absolute methanol in ratio (1/10) at room temperature for 72 h. The macerate was filtered using Watman N° 1 filter paper. The obtained filtrate was concentrated to dryness under reduced pressure at 40 °C using a rotary evaporator. The extract is stored at 4 °C until the experiments were performed [13].

### 2.2. Chemicals

2,2-azinobis-3-ethylbenzothiazoline-6-sulfonic acid (ABTS), 1,1-Diphenyl-2-picrylhydrazyl (DPPH), Potassium ferricyanide [K_3_Fe(CN)_6_], Trichloroacetic acid (C_2_HCl_3_O_2_), Ferric chloride (FeCl_3_), Sodium phosphate (NaH_2_PO_4_), Ammonium molybdate ((NH_4_)_2_MoO_4_), Sulfuric acid, Folin–Ciocalteu, Aluminium chloride (AlCl_3_), Sodium carbonate (Na_2_CO_3_), Gallic acid, Quercetin, Ascorbic acid, Butylated hydroxytoluene (BHT), Butylated hydroxyanisole (BHA), α-tocopherol were obtained from Sigma Aldrich (St. Louis, MO, USA). All the chemicals used, including the solvents, were of analytical grade. 

#### 2.2.1. Determination of Total Phenolic Content (TPC)

Total phenolic content of crude methanolic extract of *C. peregrinum* was determined by the Folin–Ciocalteu method adopted by Tanguy [14]. A volume of 300 µL of extract solution (1 mg/mL in methanol) was mixed with 1500 µL of Folin–Ciocalteu reagent (diluted 10 fold). After 4 min, 1200 µL of Na_2_CO_3_ (75 g/L) was added. The mixture was incubated at room temperature in the dark for 2 h. The absorbance of the reaction mixture was measured at 765 nm with UV/VIS spectrophotometer. Gallic acid was used as a standard for calibration curve and results were expressed as Gallic acid equivalents (μg GAE/mg). 

#### 2.2.2. Determination of Total Flavonoid Content (TFC)

Total flavonoid content of crude methanolic extract of *C. peregrinum* was performed by aluminium chloride colorimetric method adopted by Djeridane et al. [15]. A volume of 1 mL of 2% AlCl_3_ ethanol solution was mixed with 1 mL of sample solution (1 mg/mL). After incubation for 10 min at room temperature, the absorbance was measured at 415 nm with UV/VIS spectrophotometer (Thermo, Waltham, MA, USA). Quercetin was used as a standard for calibration curve and the results were expressed as Quercetin equivalents (μg QE/mg).

### 2.3. In Vitro Antioxidative Activity 

#### 2.3.1. DPPH Radical Scavenging Assay

Free radical scavenging activity of crude methanolic extract of *C. peregrinum* was determined spectrophotometrically according to the modified Blois methods [16]. Forty microliters of crude methanolic extract and standards (BHT, BHA) at various concentrations (12.5–800 μg/mL) were added to 160 µL of a methanol solution of DPPH (0.4 M) in a 96 well plate. The mixture was vigorously shaken and then incubated at room temperature for 30 min in the dark. The absorbance of the mixture was measured at 517 nm with UV/VIS spectrophotometer. The scavenging activity on the DPPH radical was expressed as inhibition percentage using the following equation:

% Inhibition = [(A_control_ − A_sample_)/A_control_] × 100

where A_control_ is the absorbance of the control reaction (containing all reagents except the test extract or standard), and A_sample_ is the absorbance of the test extract or standard.

#### 2.3.2. ABTS Radical Scavenging Assay

The ABTS radical scavenging activity was determined according to the method adopted by Re et al. [17]. The stock solution which was allowed to stand in the dark for 12 h at room temperature contained equal volumes of 7 mM ABTS salt and 2.4 mM potassium persulfate. The resultant ABTS^+^ solution was diluted with ethanol until an absorbance of 0.708 ± 0.025 at 734 nm was obtained. Forty microliters of varying concentrations (12.5–800 μg/mL) of the crude methanolic extract and standards (BHT, BHA) were allowed to react with 160 μL of the ABTS^+^ for 10 min. The absorbance of mixture was recorded at 734 nm with UV/VIS spectrophotometer.

The scavenging activity on the ABTS^+^ radical was expressed as inhibition percentage using the following equation:

% Inhibition = [(A_control_ − A_sample_)/A_control_] × 100

where A_control_ is the absorbance of the control reaction (containing all reagents except the test extract or standard), and A_sample_ is the absorbance of the extract or standard.

#### 2.3.3. Reducing Power Assay

This assay was performed following the method of Oyaizu [18]. A volume of 1 mL in different concentrations of crude methanolic extract and standards (BHA, α-tocopherol) at various concentrations (3.125–200 μg/mL) and 0.75 mL of distilled water were mixed with 1 mL of 0.2 M sodium phosphate buffer (pH 6.6) and, a volume of 1 mL of potassium ferricyanide (1%) was added to the mixture. Following incubation at 50 °C for 20 min, the reaction mixture was acidified with 1 mL of trichloroacetic acid (10%) and 0.25 mL of FeCl_3_ (0.1%) was added to this solution. The absorbance was measured at 700 nm with UV/VIS spectrophotometer.

#### 2.3.4. Total Antioxidant Capacity by Phosphomolybdenum Method

Total antioxidant capacity (TAC) of the crude methanolic extract was evaluated by the phosphomolybdenum method according to the procedure described by Prieto et al. [19]. An aliquot 300 μL (2 mg/mL) of crude methanolic extract was added in 3 mL of reagent solution containing (0.6 M sulfuric acid, 28 mM sodium phosphate and 4 mM ammonium molybdate). The mixture solution was subjected to incubation in a water bath at 95 °C for 90 min. The absorbance was measured at 695 nm with UV/VIS spectrophotometer. The total antioxidant capacity was expressed as Ascorbic acid equivalents (μg AAE/mg).

### 2.4. Photoprotective Activity

The photoprotective activity of crude methanolic extract was measured by determination in vitro of sun protection factor (SPF), which is considered to be one of the most frequently used indicators for the classification of protection levels afforded by sunscreen products against sunburn due mainly to harmful UV-B radiation [20]. In order to perform the photoprotective activity of *C. peregrinum* aerial parts, the crude methanolic extract was diluted in absolute methanol to obtain a concentration of 2 mg/mL. Spectrophotometric scanning was performed at wavelengths between 290 and 320 nm, with intervals of 5 nm with UV/VIS spectrophotometer. The readings were performed using 1 cm quartz cell and methanol used as a blank. SPF value was obtained according to the equation developed by Mansur et al. [21]:

$$\mathrm{SPF}=\mathrm{CF}\;\sum_{290}^{320}\mathrm{EE}\;\left(\lambda \right)\;I\;\left(\lambda \right)\;A\;\left(\lambda \right)$$

where EE(λ) is the erythemal effect spectrum; I(λ) is the solar intensity spectrum; Abs(λ) is the absorbance; and CF is the correction factor (=10).

The values of EE(λ) × I(λ) (Table 1) are constants, and they were determined by Sayre et al. [22].

### 2.5. Statistical Analysis

All data were expressed as means ± SD for at least three replications for each prepared sample. Statistical analysis was performed using one-sample *t*-test. The results are considered to be significant when *p* < 0.05.

## 3. Results

### 3.1. Total Phenolic and Flavonoid Content Evaluation

The total phenolic content of the crude methanolic extract of *C. peregrinum* was estimated by Folin–Ciocalteu reagent and expressed in gallic acid equivalents (GAE) and it was calculated from the linear regression equation of standard curve (y = 0.0143x + 0.2567, *R*^2^ = 0.9929). The results showed that the crude methanolic extract was found to contain a high amount of phenols (74.06 ± 1.23 μg GAE/mg).

The total flavonoid content of crude methanolic extract was determined via aluminum chloride colorimetric method and it was calculated from the linear regression equation of standard curve of quercetin (y = 0.0225x + 0.0078, *R*^2^ = 0.9965) and expressed as quercetin equivalent per gram of plant extract. The tested extract was found to contain high amounts of flavonoids (44.09 ± 2.13 μg QE/mg).

### 3.2. Antioxidant Activity

The antioxidant activity is a complex process, usually happening via several mechanisms. Thus evaluation of plant extracts antioxidant activity must be carried out by more than one test [23]. DPPH free radical scavenging, ABTS free radical scavenging, ferric-reducing power and total antioxidant capacity assays were used to evaluate the in vitro antioxidant effect of crude methanolic extract of *C. peregrinum*.

#### 3.2.1. DPPH Radical Scavenging Assay 

DPPH free radical scavenging activity of crude methanolic extract is shown in (Table 2). The results reveal that the percentage inhibition of DPPH radical was found to be increased with concentration. The methanolic extract showed maximum activity of 85.21 ± 0.67% at 100 μg/mL; whereas positive standards, BHT and BHA exhibited 94.00 ± 0.31% and 84.18 ± 0.10% inhibition respectively. The IC_50_ value of the extract was determined, and which is defined as the concentration of the extract required to scavenge 50% of DPPH free radical. Low IC_50_ value represents high antioxidant activity. In this study the IC_50_ values of extract and standards (BHT, BHA) were: 48.68 ± 1.71 μg/mL, 12.99 ± 0.41 μg/mL and 6.14 ± 0.41 μg/mL. The results showed that the methanolic extract has a good DPPH radical scavenging effect.

#### 3.2.2. ABTS Radical Scavenging Assay

In case of ABTS assay the experimental data (Table 3) reveal that the crude methanolic extract was found to be effective in scavenging the ABTS^+^ radical. The percentage inhibition of ABTS^+^ radical was concentration-dependent. The scavenging effect was 91.91 ± 0.28% at higher concentrations 800 µg/mL; whereas positive standards BHT and BHA exhibited 96.68 ± 0.39%, 95.86 ± 0.10% inhibition respectively. The IC_50_ values of the extract and the standards BHT and BHA were found to be: 63.62 ± 0.66, 1.29 ± 0.30 and 1.81 ± 0.10 µg/mL, respectively, this suggests that the tested extract has a weak ABTS^+^ free radical scavenging activity when compared with standards.

#### 3.2.3. Reducing Power Assay

The reducing power of the crude methanolic extract was evaluated using ferric to ferrous reducing activity as determined spectrophotometrically from the formation of Perl’s Prussian blue colour complex [24]. In our study, the reducing power of the methanolic extract was compared with α-Tocopherol and BHA. The results are shown in (Table 4). The results showed that the reducing power activity of the crude methanolic extract increases with the increase in concentrations of the extract solutions. The methanolic extract exhibited (0.94 ± 0.02) average absorbance at 700 nm in 200 μg/mL concentration; whereas the positive standards α-Tocopherol and BHA exhibited (1.81 ± 0.09) (1.86 ± 0.46) average absorbance in the same concentrations. 

#### 3.2.4. Total Antioxidant Capacity by Phosphomolybdenum Method

Total antioxidant capacity of crude methanolic extract of *C. peregrinum* was evaluated by the phosphomolybdenum method and was expressed as ascorbic acid equivalent per mg of plant extract. The results reveal that the crude methanolic extract was found to possess strong total antioxidant capacity (130.91 ± 4.40 μg AAE/mg).

### 3.3. Photoprotective Activity

The photoprotective activity of the crude methanolic extract was evaluated by calculation of SPF; the SPF parameter was determined by the spectrophotometric method developed by Mansur et al. [21], using the UV-B region, considered to be the region of greatest incidence during the day in which people are exposed for longer [10], in this way it has been reported that in SPF ratings, SPF values 2–12, 12–30 and ≥30 are considered as having respectively minimum, moderate and high sun protective activity [25]. As shown in experimental data (Table 5) the extract showed high absorbance values ranged between 3.45 and 3.58 at 290–320 nm, and the SPF value was found to be 35.21 ± 0.18. The results indicate that the methanolic extract have displayed high photoprotective activity.

## 4. Discussion

In the present study, phenolic and flavonoid composition as well as antioxidant and photoprotective activities of *Capnophyllum peregrinum* were investigated the first time in an attempt to unravel new possible uses of this plant. Concerning total phenolic and flavonoid contents, the results suggest that phenolics and flavonoids are important components of the crude methanolic extract. These results are in accordance with previous findings reported in other Apiaceae species extracted with methanol such as *Prangos ferulacea* Lindl, *Prangos meliocarpoides* Boiss and *Prangos uechtritzii* Boiss. & Hausskn [26], *Pituranthos chloranthus* Benth and Hook [13]. Because of polyphenols are linked to the cell-wall matrix through a glycosidic/ester linkage, their water solubility may be reduced [27]. Thus several authors displayed that alcohols are suitable solvents for the extraction of phenolic and flavonoids compounds. For example, methanol or ethanol are mostly used for the separation of this class of natural products [28] according to their best solubility in alcohols, particularly in methanol [13]. Also, it has been observed that methanolic extracts of medicinal plants exhibited high phenolic and flavonoids contents as well as antioxidant activities more than water, ethyl acetate, chloroform and acetone extracts [13,29,30]. From literature, it has been well noted that medicinal plants with high amounts of phenols and flavonoids have potent antioxidant actions [31,32,33,34]. This may explain the antioxidant activity of *C. peregrinum*. In term of DPPH-scavenging potential of methanolic extract, our results are in accordance with those of previous works, indicating close relationship between DPPH radical scavenging effect and total phenolic and flavonoid contents of Apiaceae species such as *Ammi majus* L. [35], *Ferula gummosa* Boiss [36], *Daucus carota* Linn [37], *Sesili libanotis* (L.) Koch, *Sesili libanotis* (L.) Koch [38], *Coriandrum sativum* [39], *Foeniculum vulgare*, *Anethum graveolens* [40]. Concerning the reducing power and the total antioxidant capacity, the effectiveness of the methanolic extract may be explained by its high phenol and flavonoid contents. In this context, the results of our investigation are in accordance with those published earlier, which mentioned that containing flavonoids and polyphenols may play an important role in reducing power [7] and they contribute significantly to the total antioxidant activity of many fruits such as red grape [41], Star Apple [42] and medicinal plants [43].

In case of photoprotective activity, it has been well mentioned that flavonoids have significant photoprotective effects because of their UV absorbing capacity and their antioxidants effects [44]. Thus, some flavonoids such as quercetin, kaempferol, galanin and apigenin have been evaluated for their photoprotective activity [9]. Recently, it is well reported that plant extracts rich in flavonoids are capable of absorbing ultraviolet light in the UV-B and UV-A regions [11]. In this context, several authors have associated the photoprotective activity of plant extracts with their flavonoids content [45,46,47]. Thus the photoprotective potential of the methanolic extract of *C. peregrinum* could be explained by its flavonoids content. Indeed, it has been reported that UV-B rays absorbed by the skin led to the production of aggressive free radicals such as (O_2_, ^1^O_2_, HO_2_, OH, ROO) [48]. Hence, the incorporation of antioxidants is now widely recommended in sunscreens. In this context, it also has been reported that the evaluation of most effective extracts for the antioxidant activity would be important for the development of more effective sunscreens [49]. Since, the methanolic extract exhibited antioxidant effects, is having a possibility to be used as sunscreen in pharmaceutics or cosmetics formulations. 

## 5. Conclusions

In conclusion, our study evaluates, for the first time, total phenolic and flavonoid contents in addition to antioxidant and photoprotective activities of aerial parts of *Capnophyllum peregrinum* (L.) Lange (Apiaceae) growing in Algeria, our findings revealed that the methanolic extract was found to have a high phenolic and flavonoid contents as well as antioxidant and photoprotective activities. Therefore, it appears to be used a sunscreen in pharmaceutical or cosmetic preparations and as a natural source of antioxidant. Also, it can be speculated that the findings of our work could make the background for further investigation of this species, especially research concerning individual phenolic compounds. 

## Figures and Tables

**Table 1:** The Normalized product function used in the calculation of sun protection factor (SPF).

Wavelength (nm)	EE × I (Nomalized)
290	0.0150
295	0.0817
300	0.2874
305	0.3278
310	0.1864
315	0.0839
320	0.0180

**Table 2:** Antioxidant activity by the 1,1-Diphenyl-2-picrylhydrazyl (DPPH) assay *C. peregrinum* methanolic extract.

Concentration μg/mL	% Inhibition in DPPH Assay		
	MeOH Extract	BHA	BHT
12.5	16.97 ± 1.06	76.55 ± 0.48	49.09 ± 0.76
25	30.26 ± 2.07	79.89 ± 0.26	72.63 ± 2.06
50	53.90 ± 1.65	81.73 ± 0.10	88.73 ± 0.89
100	85.21 ± 0.67	84.18 ± 0.10	88.73 ± 0.89
200	86.63 ± 0.51	87.13 ± 0.17	94.97 ± 0.08
400	86.92 ± 0.17	89.36 ± 0.19	95.38 ± 0.41
800	86.46 ± 0.19	90.14 ± 0.00	95.02 ± 0.23
**IC50 μg/mL**	**48.68 ± 1.71**	**6.14 ± 0.41**	**12.99 ± 0.41**

**Table 3:** Antioxidant activity by the 2,2-azinobis-3-ethylbenzothiazoline-6-sulfonic (ABTS) assay *C. peregrinum* methanolic extract.

Concentration μg/mL	% Inhibition in ABTS Assay		
	MeOH Extract	BHA	BHT
12.5	5.01 ± 0.93	69.21 ± 0.40	92.83 ± 1.42
25	7.22 ± 0.47	78.23 ± 1.34	94.68 ± 0.42
50	13.85 ± 0.09	88.12 ± 1.28	94.95 ± 0.90
100	28.30 ± 0.37	88.76 ± 3.07	95.32 ± 0.25
200	42.75 ± 0.43	90.85 ± 1.74	95.59 ± 0.47
400	68.36 ± 0.09	90.95 ± 0.51	95.83 ± 0.15
800	91.91 ± 0.28	96.68 ± 0.39	95.86 ± 0.10
**IC50 μg/mL**	**63.62 ± 0.66**	**1.29 ± 0.30**	**1.81 ± 0.10**

**Table 4:** Antioxidant activity by the reducing power assay of *C. peregrinum* methanolic extract.

Concentration μg/mL	Absorbance		
	MeOH Extract	BHA	α-Tocopherol
3.125	0.08 ± 0.01	0.28 ± 0.02	0.11 ± 0.00
6.25	0.09 ± 0.01	0.54 ± 0.02	0.16 ± 0.00
12.5	0.13 ± 0.01	0.75 ± 0.07	0.21 ± 0.03
25	0.19 ± 0.02	1.28 ± 0.28	0.35 ± 0.03
50	0.29 ± 0.03	1.49 ± 0.11	0.73 ± 0.03
100	0.58 ± 0.04	1.79 ± 0.16	1.37 ± 0.08
200	0.94 ± 0.02	1.86 ± 0.46	1.81 ± 0.09

**Table 5:** Photoprotective activity of *C. peregrinum* methanolic extract.

Wavelength (nm)	EE × I	Absorbance
290	0.0150	3.583 ± 0.011
295	0.0817	3.561 ± 0.026
300	0.2874	3.53 ± 0.011
305	0.3278	3.52 ± 0.036
310	0.1864	3.484 ± 0.02
315	0.0839	3.478 ± 0.039
320	0.0180	3.453 ± 0.03
SPF = CF$\;\sum_{290}^{320}\mathrm{EE}\;\left(\lambda \right)\;I\;\left(\lambda \right)\;A\;\left(\lambda \right)$ = 35.21 ± 0.18		
